# MOB-mediated regulation of septation initiation network (SIN) signaling is required for echinocandin-induced hyperseptation in *Aspergillus fumigatus*

**DOI:** 10.1128/msphere.00695-23

**Published:** 2024-02-13

**Authors:** Harrison I. Thorn, Xabier Guruceaga, Adela Martin-Vicente, Ashley V. Nywening, Jinhong Xie, Wenbo Ge, Jarrod R. Fortwendel

**Affiliations:** 1 Graduate Program in Pharmaceutical Sciences, College of Pharmacy, University of Tennessee Health Science Center, Memphis, Tennessee, USA; 2 Department of Clinical Pharmacy and Translational Science, College of Pharmacy, University of Tennessee Health Science Center, Memphis, Tennessee, USA; 3 Integrated Program in Biomedical Sciences, College of Graduate Health Sciences, University of Tennessee Health Science Center, Memphis, Tennessee, USA; 4 Department of Microbiology, Immunology, and Biochemistry, College of Medicine, University of Tennessee Health Science Center, Memphis, Tennessee, USA; 5 Department of Pharmacy and Pharmaceutical Sciences, St. Jude Children’s Research Hospital, Memphis, Tennessee, USA; University of Guelph, Guelph, Canada

**Keywords:** *Aspergillus fumigatus*, septation, echinocandin, virulence determinants, cell wall

## Abstract

*Aspergillus fumigatus* is a major invasive mold pathogen and the most frequent etiologic agent of invasive aspergillosis. The currently available treatments for invasive aspergillosis are limited in both number and efficacy. Our recent work has uncovered that the β-glucan synthase inhibitors, the echinocandins, are fungicidal against strains of *A. fumigatus* with defects in septation initiation network (SIN) kinase activity. These drugs are known to be fungistatic against strains with normal septation. Surprisingly, SIN kinase mutant strains also failed to invade lung tissue and were significantly less virulent in immunosuppressed mouse models. Inhibiting septation in filamentous fungi is therefore an exciting therapeutic prospect to both reduce virulence and improve current antifungal therapy. However, the SIN remains understudied in pathogenic fungi. To address this knowledge gap, we characterized the putative regulatory components of the *A. fumigatus* SIN. These included the GTPase, SpgA, it’s two-component GTPase-activating protein, ByrA/BubA, and the kinase activators, SepM and MobA. Deletion of *spgA*, *byrA,* or *bubA* resulted in no overt septation or echinocandin susceptibility phenotypes. In contrast, our data show that deletion of *sepM* or *mobA* largely phenocopies disruption of their SIN kinase binding partners, *sepL* and *sidB*, respectively. Reduced septum formation, echinocandin hypersusceptibility, and reduced virulence were generated by loss of either gene. These findings provide strong supporting evidence that septa are essential not only for withstanding the cell wall disrupting effects of echinocandins but are also critical for the establishment of invasive disease. Therefore, pharmacological SIN inhibition may be an exciting strategy for future antifungal drug development.

**IMPORTANCE:**

Septa are important structural determinants of echinocandin susceptibility and tissue invasive growth for the ubiquitous fungal pathogen *Aspergillus fumigatus*. Components of the septation machinery therefore represent promising novel antifungal targets to improve echinocandin activity and reduce virulence. However, little is known about septation regulation in *A. fumigatus*. Here, we characterize the predicted regulatory components of the *A. fumigatus* septation initiation network. We show that the kinase activators SepM and MobA are vital for proper septation and echinocandin resistance, with MobA playing an essential role. Null mutants of *mobA* displayed significantly reduced virulence in a mouse model, underscoring the importance of this pathway for *A. fumigatus* pathogenesis.

## INTRODUCTION

Life-threatening fungal infections can be experienced secondary to immunosuppression in treatment regimens such as chemotherapy and transplant rejection prevention, and are on the rise as increased incidence of autoimmune diseases drive the use of immunosuppressive drugs (1). Opportunistic fungal pathogens take advantage of this vulnerable state to cause a wide range of diseases. Invasive pulmonary aspergillosis (IPA) is one of the most common of these infections caused by filamentous fungi and remains a scourge in immunocompromised populations, maintaining a mortality rate as high as 50% (2). IPA is caused foremost by *Aspergillus fumigatus*, a ubiquitous mold ascomycete. It is estimated that hundreds of *A. fumigatus* conidia are inhaled by each person daily and can cause disease when pulmonary leukocytes fail to effectively clear them (3). Under these circumstances, *A. fumigatus* colonizes and invades the lung tissue, causing massive tissue damage. Unfortunately, treatment options for IPA remain limited. Only three classes of antifungal drugs are currently Federal Drug Administration (FDA) approved to treat these infections: the polyenes, the triazoles, and the echinocandins (3). Amphotericin B, the only polyene used for these infections, has significant associated hepatotoxicity (4).Triazoles are the first-line treatment for IPA and are highly effective against *Aspergillus* infections, but unfortunately, a combination of toxicity risks and emerging resistance in clinical and environmental isolates puts the efficacy of triazoles at risk (4). Finally, echinocandins have been used in salvage or combination therapies for invasive aspergillosis. However, these compounds are considered fungistatic against *Aspergillus*, requiring re-establishment of normal immune function for pathogen clearance (5, 6). Therefore, the need for novel therapies is urgent.

Previous work in our laboratory uncovered *A. fumigatus* mutants of the septation initiation network (SIN) kinases, SepH, SepL, and SidB, as hypersusceptible to echinocandins, converting this drug class from static to cidal (7). Surprisingly, we also found that these aseptate mutant strains were avirulent in mouse models of IPA (7). These findings suggest that pursuing pharmacological avenues to block septation in *A. fumigatus* could result in highly efficient therapeutic approaches that both inhibit pathogenic growth and improve anti-*A*. *fumigatus* echinocandin activity. Unfortunately, our previous work represents the only characterization of the role of the SIN pathway in the support of *A. fumigatus* pathogenesis and echinocandin resistance. Therefore, we set out to characterize regulation of SIN kinase signal transduction in *A. fumigatus*.

The SIN is a tripartite kinase cascade and has been most extensively studied in the model fission yeast, *Schizosaccharomyces pombe,* where the core signaling kinases are Cdc7p, Sid1p, and Sid2p (8). The *S. pombe* SIN is driven by activation of a fungus-specific GTPase, Spg1p, at the spindle pole body (9). Due to the fundamental coupling of septation and cytokinesis for yeast cell growth, Spg1 is essential in this model yeast (9). The activation status of Spg1p is directly regulated by a two-component GTPase-activating protein (GAP) composed of the Byr4p and Cdc16p proteins (10). As the Spg1 GAP proteins, Byr4 and Cdc16, function as negative regulators of septation in *S. pombe*, mutations in either result in multiseptated yeast cells (11, 12). Septation signals are transduced through Spg1p-mediated phosphorylation of Cdc7p by an upstream kinase which then leads to the subsequent phosphorylation of the kinases Sid1p and Sid2p in a signaling cascade (9, 13–15). Following activation, Sid2p has been shown to localize to the site of septation along with its regulator Mob1p (16). The *S. pombe* Cdc14p (a Sid1p regulator) and Mob1p proteins are each essential for septation (14, 16, 17). Although orthologs of Spg1, Byr4, Cdc16, Cdc14, and Mob1 are present in *A. fumigatus*, each of these regulatory components remain unstudied in relation to *A. fumigatus* septation, echinocandin stress resistance, and virulence.

Here, we characterize for the first time the putative *A. fumigatus* SIN regulatory components, SpgA, ByrA, BubA, SepM, and MobA. BLAST searches of the *A. fumigatus* genome reveal these as orthologs of the *S. pombe* pathway components, Spg1, Byr4, Cd16, Cdc14, and Mob1, respectively. We show that deletion of *spgA*, *byrA,* or *bubA* does not cause a discernable septation defect or an increase in echinocandin susceptibility. Furthermore, loss of the putative GTPase, *spgA,* and GAP components, *byrA* and *bubA*, resulted in only a modest growth defect. In contrast, loss of *sepM* or *mobA* resulted in greatly impaired septation, though mutant hyphae were able to form infrequent septa in mature cultures. The *sepM* and *mobA* deletion mutants displayed increased susceptibility to cell wall stress, increased susceptibility to echinocandins, and significantly decreased virulence in a corticosteroid mouse model of IPA. Taken together, our data emphasize the necessity of septa to withstand cell wall stress and provide further evidence supporting septation inhibition as a strategy for novel antifungal development (7, 18, 19).

## RESULTS

### Deletion of the SIN kinase regulators, *sepM* or *mobA*, phenocopies loss of SIN kinase function

**Fig 1:**
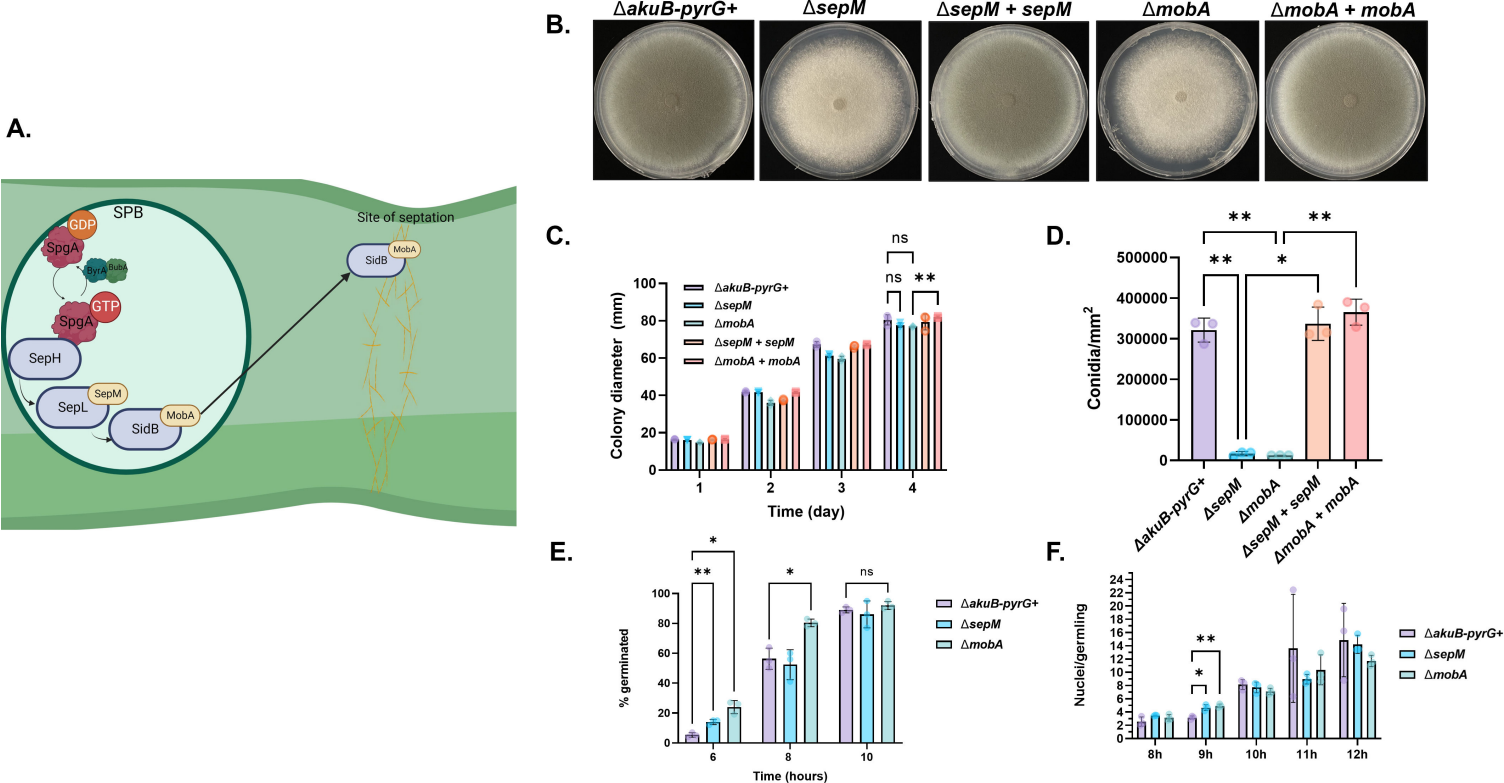
Deletion of the putative SIN regulators, *sepM* and *mobA*, alters colony morphology and early development. (**A**) Schematic of the proposed *A. fumigatus* SIN kinase cascade. The initiating kinase, SepH, receives activating signals from the fungus-specific GTPase, SpgA, which is regulated by the two-component GAP comprised of BubA and ByrA. SepH then activates SepL in a SepM-dependent manner which, in turn, activates SidB in a MobA-dependent manner. Schematic was made using Biorender. (**B**) Colony morphology of the control strain (*akuB-pyrG+*), the *sepM* deletion mutant (∆*sepM*), the Δ*sepM* complemented strain (∆*sepM +sepM*), the *mobA* deletion mutant (∆*mobA*), and the Δ*mobA* complemented strain (∆*mobA +mobA*). Ten thousand conidia were spot-inoculated onto the center of glucose minimal media agar and allowed to grow for 4 days at 37°C. (**C**) Quantitation of colony diameter for each day post-inoculation. Colonies were grown as explained above for 4 days. Colony diameter was measured with calipers at the end of each 24-h period. Data presented are the average of three biological replicates per strain ± SD. Data were analyzed by two-way analysis of variance (ANOVA) with Turkey’s multiple comparison test. (**D**) Quantitative comparison of conidiation among all strains. Each strain was cultured as described above. On day 4, colony area (mm^2^) was calculated for each culture and conidia were harvested in sterile distilled water. Conidia were counted via hemocytometer. Data are presented as total number of conidia normalized to total colony area ± SD. Data were analyzed by one-way ANOVA with Dunnett’s multiple comparison’s test. (**E**). Coverslip cultures were grown for each strain in triplicate for the indicated time. Each data point represents a different culture. After the indicated time point, coverslips were washed with phosphate buffered saline (PBS), fixed with a solution of 5% formalin, 2% triton-X by volume, treated with RNAse A, and stained with propidium iodide fluorescently stain nuclei. Between 60 and 100 germlings were evaluated for presence of one or more septa and number of nuclei. The average number of germlings with one or more septa and average number of nuclei per germling are shown. Data were analyzed by two-way ANOVA using Dunnett’s multiple comparisons test. (**F**) Coverslip cultures were grown for each strain in triplicate for the indicated time. Each data point represents a different culture. At each time point, coverslips were visualized by light microscopy and 200–400 conidia/germlings were evaluated for polarity establishment. The percentage of germlings with established polarity is shown. Data were analyzed by unpaired *t*-tests. All data in (**B–F**) represent three biological replicates. Error bars represent standard deviation. * = *P* < 0.05. ** = *P* < 0.01.

To assess if the components functioning at the apex of the putative *A. fumigatus* SIN (Fig. 1A) are required for septation and echinocandin resistance, deletion mutants of the orthologs of *S. pombe spg1* (*spgA*) (AFUB_076380), byr4 (*byrA*) (AFUB_052950), and *cdc16* (*bubA*) (AFUB_003320) were generated in the wild-type CEA10 background using CRISPR/Cas9 editing (Fig. S1) (20). As septation-defective mutants often display developmental defects, a radial growth assay was first conducted to assess colony development in the resulting mutants. After 4 days of incubation on minimal media at 37°C, the *bubA* and *byrA* deletion mutants (Δ*bubA* and Δ*byrA*, respectively) developed colonies with a diameter that was only ~60% the size of the wild type (Fig. S3A and B). Calcofluor white (CFW) staining was employed to assess the septation status in mature hyphae of the three mutant strains, revealing no overt septation defect relative to the parental CEA10 (Fig. S2A). In addition, drug strip diffusion assays revealed no changes in susceptibility to micafungin (MFG) (Fig. S3C). Taken together, we conclude that SpgA, ByrA, and BubA do not contribute to septation regulation in a manner that significantly impacts echinocandin resistance.

Activation of the penultimate SIN kinase, SepL, and the terminal SIN kinase, SidB, is putatively dependent on conserved interacting proteins, SepM and MobA, that are uncharacterized in *A. fumigatus* (Fig. 1A). To analyze the impact of putative SIN kinase regulatory proteins on septation, echinocandin resistance, and virulence, we next generated deletion mutants of the *A. fumigatus* orthologs of the *S. pombe* Cdc14 kinase binding protein, SepM (AFUB_012650), and the Mob1 kinase binding protein, MobA (AFUB_028050). These studies employed the Δ*akuB-pyrG*^+^ parental strain, a prototrophic derivative of the ∆*akuB* strain originally generated in the A1163 (CEA10) genetic background (20, 21). To ensure phenotypes were the result of targeted gene deletion, gene complementation was carried out by re-incorporating the *sepM* or *mobA* coding sequence into the native locus of each deletion mutant strain. Therefore, expression of the re-inserted gene was under the control of the endogenous promoter (Fig. S1). Impacts of *sepM* (Δ*sepM*) or *mobA* (Δ*mobA*) loss on colony development were first determined through a radial growth assay on minimal media. After 4 days of incubation at 37°C, there was no statistically significant difference in colony diameter between the control (Δ*akuB-pyrG*+) and manipulated strains (Fig. 1B and C). Notably, the Δ*sepM* and Δ*mobA* mutants developed non-pigmented colonies compared to the normal green pigmentation expected during *A. fumigatus* development (Fig. 1B). As the green coloration typically represents pigmented conidia formation, this suggested a potential defect in conidia production or conidia pigmentation due to the absence of either *sepM* or *mobA*. To test if the colony coloration of Δ*sepM* and Δ*mobA* was due to a pigmentation defect, 1.5 * 10^8^ conidia of the control strain, both deletion mutants and both complemented strains were pelleted to evaluate their coloration (Fig. S2). All pellets appeared to be the same color, suggesting the coloration on minimal media is not due to a pigmentation defect. To test if deletion of *sepM* or *mobA* caused deficient conidia production, 4-day-old colonies were harvested to quantify conidia production. The Δ*sepM* and Δ*mobA* deletion strains were determined to produce nearly 10-fold fewer conidia than the control strain or their complements, Δ*sepM + sepM* and Δ*mobA + mobA*, respectively (Fig. 1D).

Septation is an early growth landmark event during *A. fumigatus* development and is typically coupled to initiation of mitosis and polarity establishment (22). To investigate if timing of polarity establishment and/or nuclear replication was dependent on *sepM* or *mobA*, germlings of the Δ*sepM* and Δ*mobA* mutants were cultured and evaluated at regular intervals for polarity establishment (i.e., formation of a germ tube) in comparison to the parental control strain. Early in the germination process (6 h post-inoculation), the Δ*sepM* and Δ*mobA* mutants displayed a modest, but statistically significant, increase in percent germinated conidia (Fig. 1E). By 8 h post-inoculation, only the Δ*mobA* mutant continued to display an increased germination rate and, by 10 h post-inoculation, no significant differences were noted (Fig. 1E). In a separate experiment, germlings of mutant and control strains cultured for up to 12 h were fixed and stained with propidium iodide (PI) at 2-h intervals to visualize the number of nuclei per germling (Fig. 1F). Beginning with the earliest timepoint that mitosis was evident (8 h post-inoculation), the average number of nuclei per germling was found to be consistent within the range of a single mitotic cycle across all strains tested. There were no statistically significant differences in the number of nuclei per germling at any timepoint (Fig. 1F). Therefore, the timing of mitosis was not disrupted by a loss of individual SIN regulatory components.

**Fig 2:**
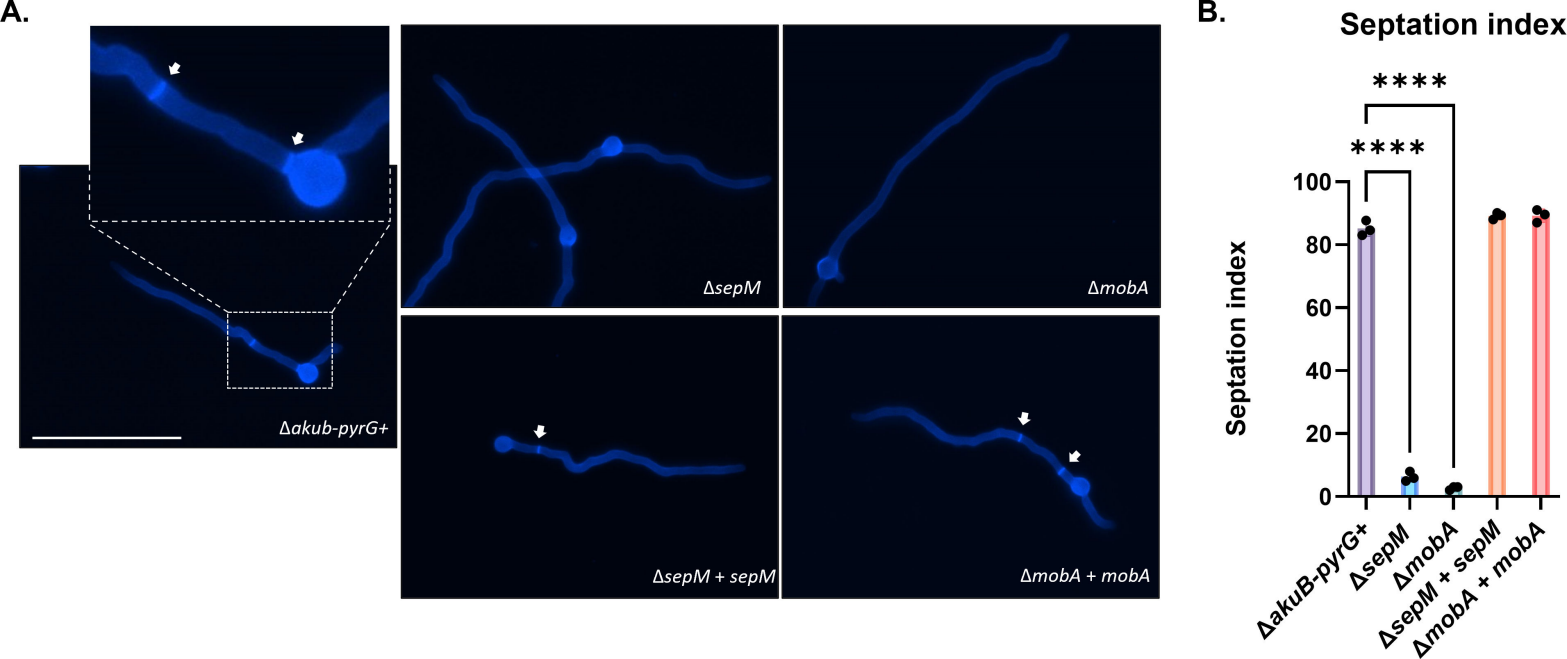
Loss of *sepM* or *mobA* results in septation deficiency. (**A**) Ultraviolet micrographs of germlings of the control and manipulated strains. Conidia from each strain were inoculated over sterile coverslips submerged in minimal media broth and incubated for 10 h at 37°C. After incubation, coverslips were removed and adhered germlings stained with calcofluor white for visualization of septa. White arrows denote septa in the control strain. Scale bars = 50 µm. (**B**) Quantitative comparison of germling septation. At least 100 germlings from each strain in (**A**) were enumerated for the presence of septa. Data are reported as the septation index which is the percent of germlings displaying at least one septum. All data points represent the average of three replicate cultures and error bars represent standard deviation. Statistical analyses were performed by one-way analysis of variance. **** = *P* < 0.0001.

To determine if deletion of either *sepM* or *mobA* also resulted in loss of septation similar to the previously reported SIN kinase mutations, we next employed (CFW) staining of germlings to visualize and quantitate septum development for the control and deletion strains. Conidia from each strain were inoculated into minimal media broth and allowed to germinate for 10 h at 37°C. After incubation, germlings were stained with CFW to visualize septa by fluorescence microscopy (Fig. 2A). The septation index, defined as the percent of germlings displaying at least one septum, was calculated for each strain (23, 24). Whereas approximately 85% of the control strain germlings had at least one septum, both the Δ*sepM* and Δ*mobA* mutants displayed significantly fewer septa, whereas no significant difference was observed between the mutant strains (Fig. 2B).

### Loss of *mobA* or *sepM* increases susceptibility to cell wall stress agents and to the echinocandin antifungal drug class

**Fig 3:**
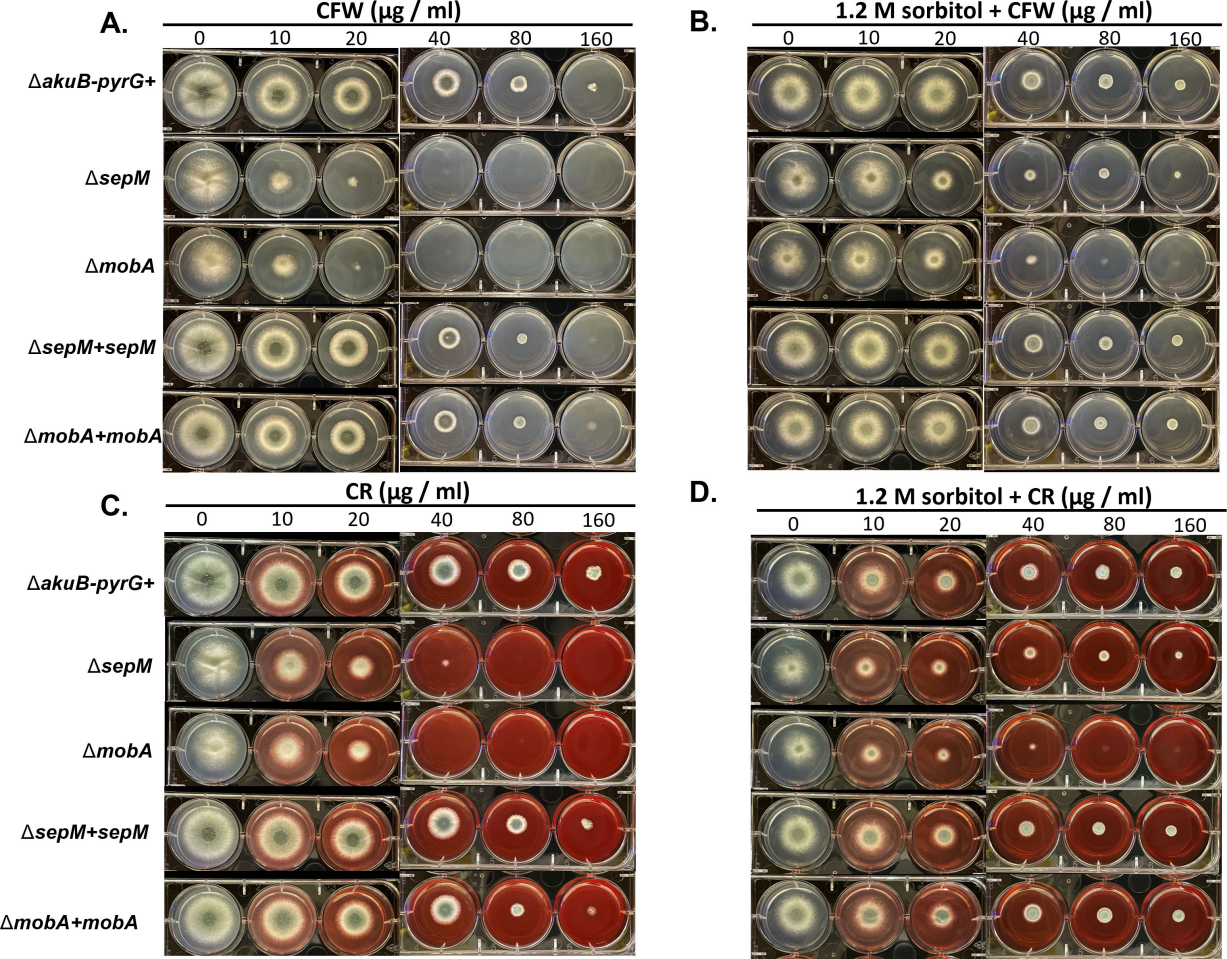
Deletion of *mobA* or *sepM* causes hypersusceptibility to cell wall stress. Six-well plates were spot inoculated with 5 µL of sterile water containing 10^6^ conidia/mL of the indicated strains. Each well of the plate contained either (**A**) glucose minimal media (GMM) containing the indicated concentration of CFW, (**B**) GMM supplemented with 1.2 M sorbitol (SMM, sorbitol minimial media) and the indicated concentration of CFW, (**C**) GMM containing the indicated concentration of CR, or (D) GMM supplemented with 1.2 M sorbitol and the indicated concentration of CR. Plates were incubated at 37°C for 2 days. Representative images of two replicates per condition are shown.

The *A. fumigatus* SIN kinase loss-of-function mutants were previously shown to be hypersusceptible to cell wall stress, likely due to the loss of the protective barrier provided by the septum (18, 19, 25). Therefore, we next sought to determine if loss of *sepM* or *mobA* also generated increased susceptibility to cell wall stress. To test this, the mutant and control strains were first cultured in the presence of increasing concentrations of the common cell wall stress agents congo red (CR) and CFW. After 2 days of incubation, the control strain and complemented strains displayed colony development under all tested conditions, with impaired growth at increasing concentrations of both agents (Fig. 3A and C). In contrast, the Δ*sepM* and Δ*mobA* mutants displayed almost complete lack of growth at concentrations above 20 µg/mL CFW and at 40 µg/mL CR (Fig. 3A and B). Growth was restored for both mutant strains in the presence of high concentrations of CFW or CR on media osmotically stabilized by supplementation with sorbitol, further supporting that CR- and CFW-mediated growth inhibition was due to cell wall stress-induced lysis (Fig. 3B and D).

**Fig 4:**
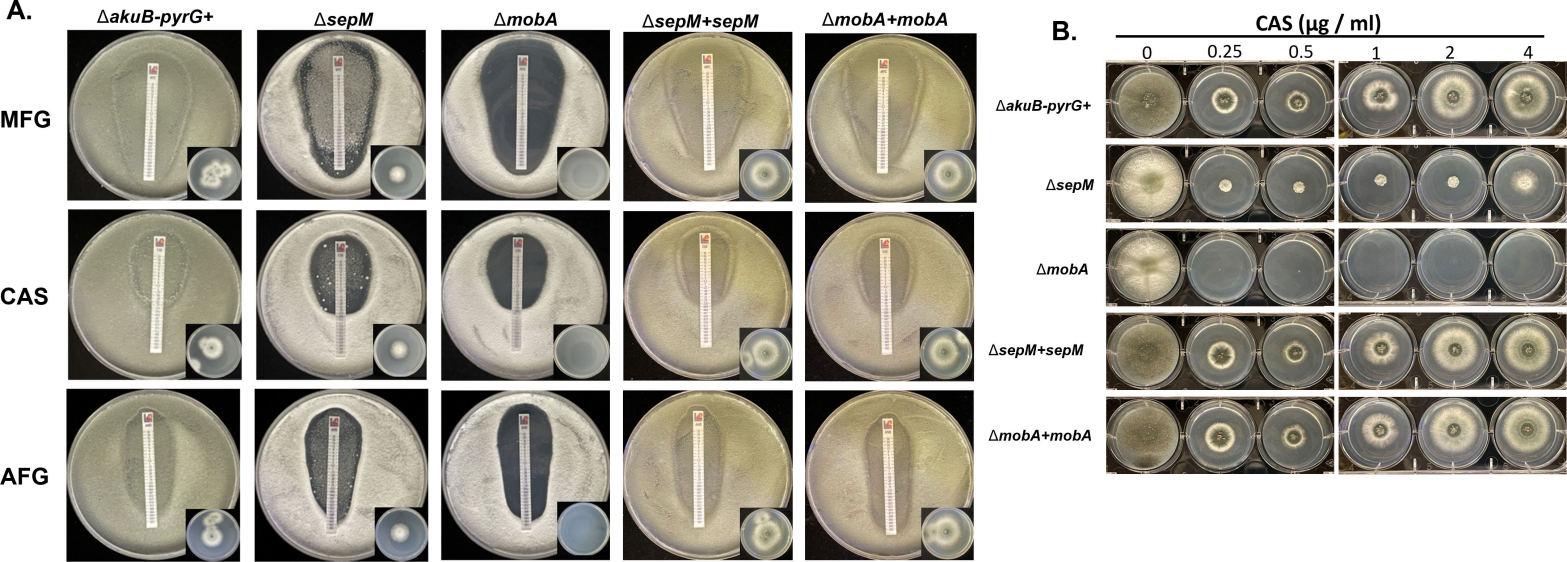
The ∆*sepM* mutant maintains low-level echinocandin resistance on solid agar. (**A**) Drug strip diffusion assays for assessment of echinocandin susceptibility. Five-hundred microliters containing 10^6^ conidia of the indicated strains was spread across glucose minimal media (GMM) agar plates and allowed to dry. Drug-embedded strips containing CAS, MFG, or AFG were then applied to the plates, which were incubated for 2 days at 37°C. Representative images from assays run in triplicate are shown. (**B**) Agar plates were spot inoculated with 5 µL of sterile water containing 10^6^ conidia/mL of the indicated strains. Each well of the plate contained GMM supplemented with the indicated concentration of CAS. Plates were incubated at 37°C for 3 days. Representative images of duplicate assays are shown.

We next tested if this cell wall stress hypersusceptibility also extended to the echinocandins, clinically relevant antifungal compounds which inhibit synthesis of the major cell wall component β-1,3-glucan (6). Using drug strip diffusion assays, the control and complemented strains displayed only a zone of depressed growth in the presence of each of the major echinocandins, MFG, caspofungin (CAS), or anidulafungin (AFG) (Fig. 4A). These findings are consistent with fungistatic echinocandin activity against *A. fumigatus* (6). In contrast, the Δ*mobA* mutant displayed clean zones of clearance for all three tested echinocandins (Fig. 4A). Agar cores taken from within the zone of clearance generated no residual growth when sub-cultured to drug-free media, suggesting that echinocandins were fungicidal against *A. fumigatus* lacking *mobA* (Fig. 4A, inset). Strikingly, the Δ*sepM* mutant continued to display patchy areas of growth within the zone of inhibition and, while not as robust as the wild type, agar cores taken from the Δ*sepM* zones of clearance for each drug were able to form colonies on drug-free media (Fig. 4A, inset). A common factor complicating the clinical use of echinocandins against *Aspergilli* is the induction of the caspofungin paradoxical effect (CPE), a phenomenon of decreased drug efficacy at increasing drug concentration (26, 27). To test if the CPE was retained in the Δ*sepM* mutant, we conducted a spot-inoculation assay using growth-inhibitory and CPE-inducing concentrations of caspofungin (28). Indeed, the control strain, complemented strains, and Δ*sepM* mutant demonstrated more robust growth at higher concentrations of the drug, indicative of an intact CPE (Fig. 4B). In contrast, the ∆*mobA* mutant did not grow at any concentration of caspofungin (Fig. 4B).

**Fig 5:**
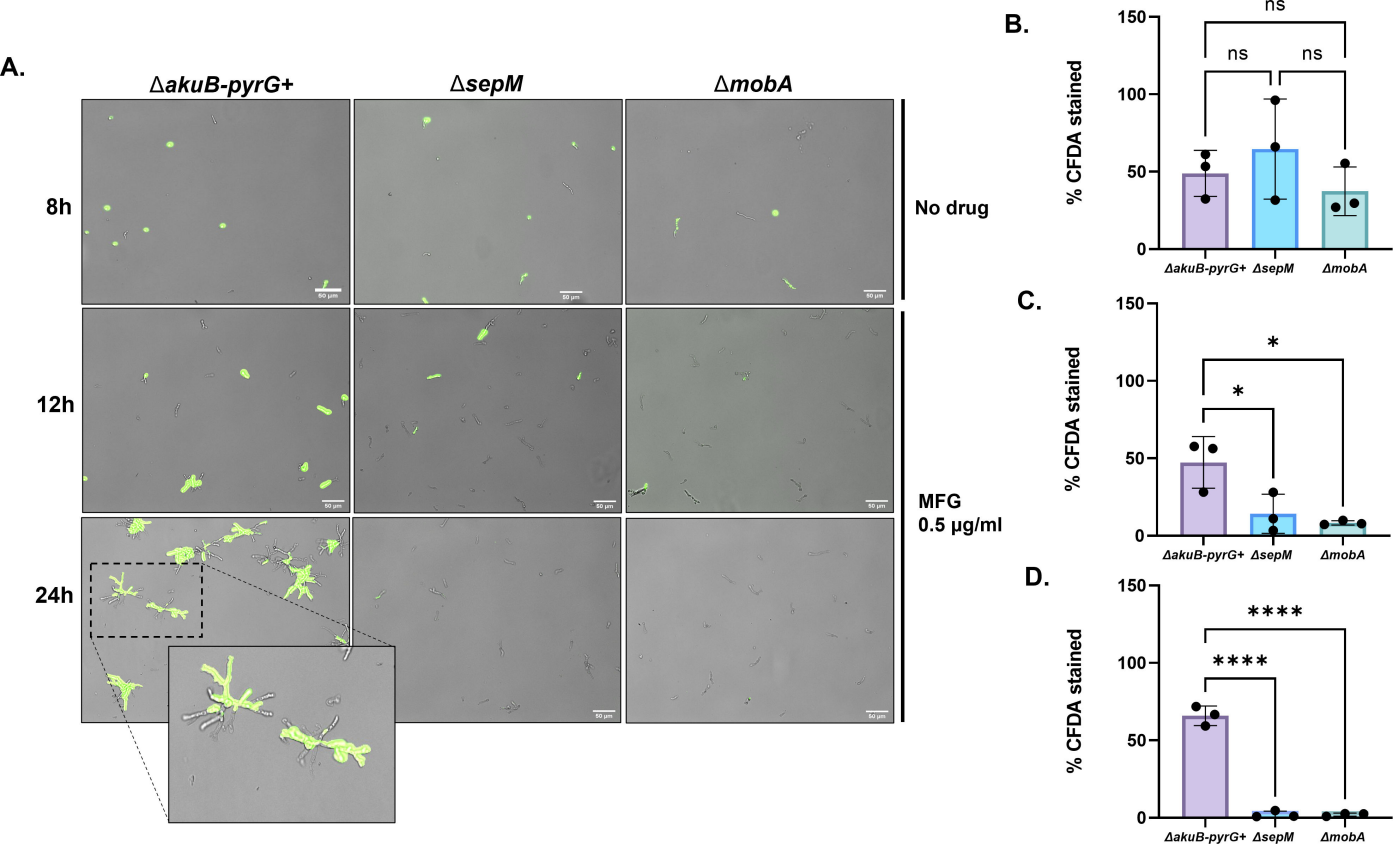
Echinocandins are fungicidal to SIN-regulator mutants in submerged culture. (**A**) Submerged cultures (with or without 0.5 µg/mL MFG) containing 10^4^ conida/mL were grown on sterilized coverslips. After the indicated timepoint, coverslips were removed from cultures, washed with phosphate buffered saline (PBS), and stained with CFDA to detect live cells. Representative merged images of bright field and CFDA fluorescence visualized with a Green Fluorescent Protein (GFP) filter are shown. Green fluorescence indicates a living colony and/or germling. (**B**) Percentage of positively stained microcolonies after 8 h of growth in drug-free minimal media. (**C**) Percentage of positive stained microcolonies after 12 h of growth in minimal media containing 0.5 µg/mL MFG. (**D**) Percentage of positively stained microcolonies after 24 h of growth in minimal media containing 0.5 µg/mL MFG. Data in (**B–D**) were analyzed by one-way analysis of variance. All error bars represent SD. * = *P* < 0.05. **** = *P* < 0.0001.

To quantitatively measure the cidal nature of echinocandin activity against the mutant strains, we next performed a viability assay using the live-staining dye carboxyfluorescein diacetate (CFDA) (6). Conidia were cultured to the germling stage (8 h) in the absence of drug as a control, and for 12 or 24 h in the presence of 0.5 µg/mL MFG (Fig. 5). In the absence of drug, no significant difference in viability (i.e., percentage of germlings staining positive with CFDA) was noted among the tested strains (Fig. 5A and B). Whereas the percent viable germlings remained unchanged for the control strain after 12 h of drug stress (47.4% ± 13.6%), viability of germlings of the *∆mobA* (8.34% ± 1.13%) and Δ*sepM* (14.2% ± 10.3%) strains was significantly decreased (Fig. 5A and C). After 24 h of growth under MFG stress, little-to-no detectable viable germlings and/or microcolonies remained in cultures of both deletion strains (Fig. 5A and D).

**Fig 6:**
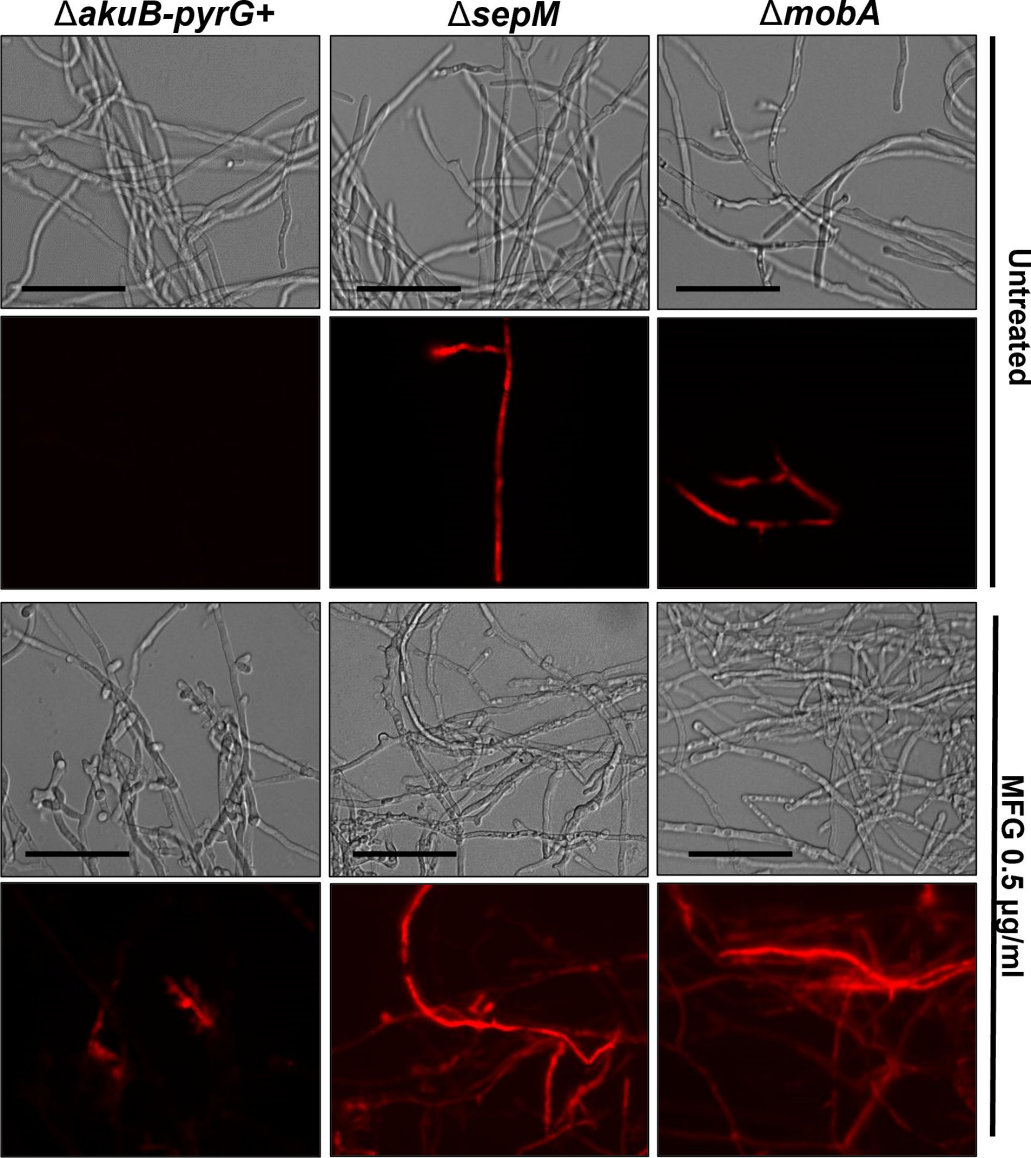
Mature hyphae of the Δ*sepM* and Δ*mobA* mutants are hypersusceptible to echinocandins. Submerged coverslip cultures containing 10^4^ conidia/mL were grown for 16 h at 37°C. Spent culture media was then replaced with fresh minimal media containing 0.5 µg/mL MFG and incubated at 37°C for an additional 2 h. Fluorescence represents hyphae which were penetrable to the nucleic acid-staining PI, suggesting hyphal death. Representative images of bright fields and fluorescence are shown. Scale bars = 50 µm.

To test if echinocandins also displayed increased activity against mature hyphae of Δ*sepM* and Δ*mobA*, the control and mutant strains were cultured for 16 h in the absence of drug and were then treated with 0.5 µg/mL MFG for 2 h. As a measure of hyphal damage, treated and untreated cultures were then stained with PI and visualized using fluorescence microscopy. Interestingly, under no drug stress, both the Δ*sepM* and Δ*mobA* strains revealed sparse positive staining with PI indicating low levels of cell permeability (Fig. 6). Under drug stress (0.5 µg/mL MFG), the control strain displayed dead hyphal segments, likely delimited by septa, but maintained integrity of the remaining hyphae (Fig. 6). This finding correlated well with the live-stain assay in Fig. 5. In contrast, both mutant strains demonstrated extensive PI staining of elongated hyphal compartments, likely due to a lack of septum-dependent protection of hyphal compartments (7, 18, 19, 29). Taken together, our findings support the hypothesis that SepM- and MobA-mediated septation is required for protection against stress induced by cell wall intercalating agents (i.e., CR or CFW) or by glucan synthesis inhibition (i.e., echinocandins). As the Δ*sepM* mutant is able to grow on solid agar impregnated with echinocandin and maintains the CPE, we also conclude that SepM is not absolutely required for survival in the presence of echinocandins.

### SepM is not required for stress-induced hyperseptation in the presence of caspofungin

**Fig 7:**
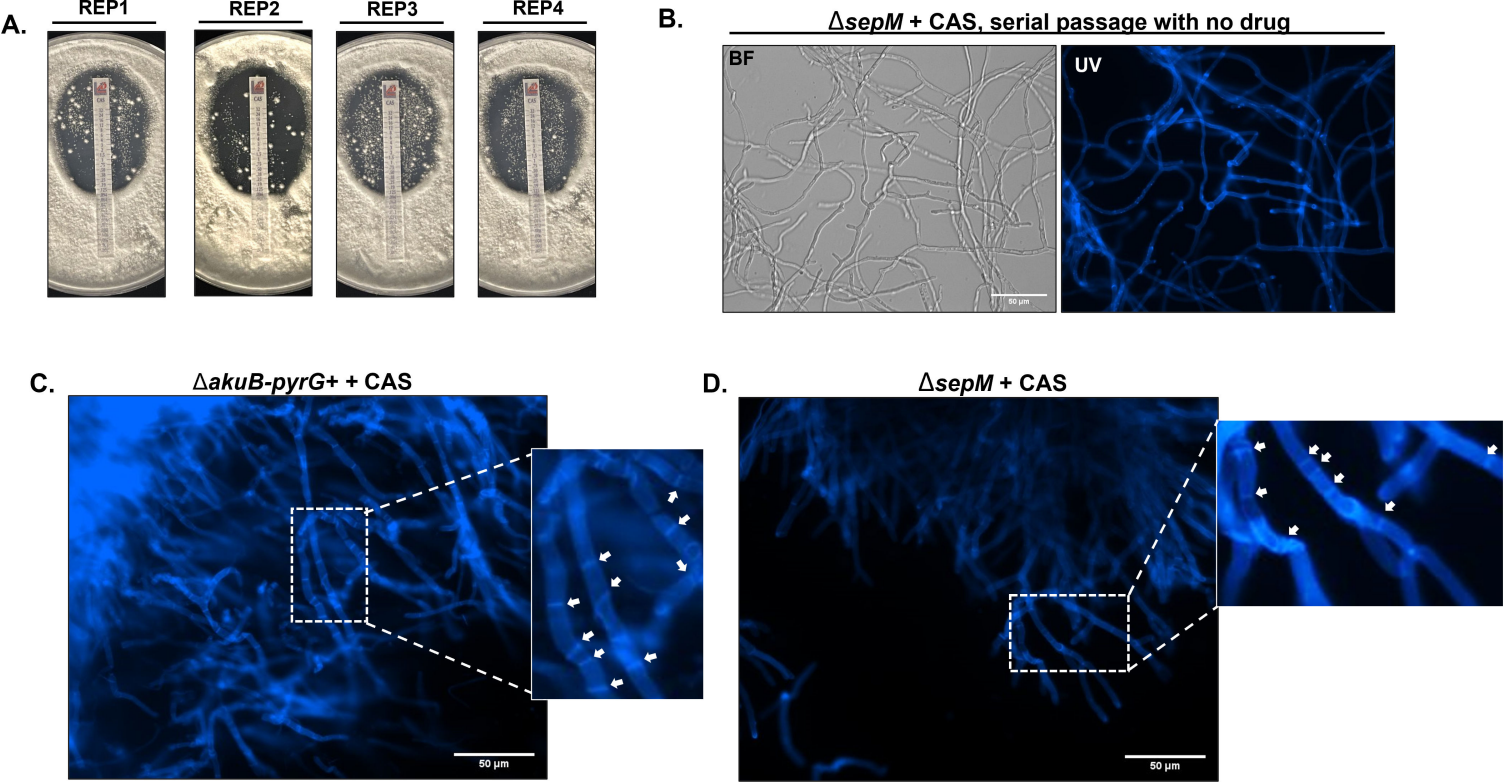
The ∆*sepM* mutant retains the capacity for inducible hyperseptation in response to caspofungin stress on solid agar. (**A**) CAS drug strip diffusion assays conducted after drug-free passage of Δ*sepM* microcolonies isolated from the zone of clearance from Fig. 4 CAS plates. REP1, REP2, REP3, and REP4 indicate four microcolony replicates isolated for analysis. (**B**) Representative micrograph from CFW-stained isolate “REP1” after a single passage under drug-free conditions. Note the continued lack of septa. (**C**) Analysis of septation in control hyphae isolated from the CAS drug strip zone of inhibition. Hyphae were stained with CFW to visualize septa and a representative micrograph field is shown. Septa are indicated with arrows. (**D**) Analysis of septation in microcolonies of Δ*sepM* hyphae isolated from the CAS drug strip zone of inhibition. Hyphae were stained with CFW to visualize septa and a representative micrograph field is shown. Septa are indicated with arrows.

We next sought to determine the mechanism underpinning continued viability of the Δ*sepM* mutant on solid media containing echinocandin. To test the hypothesis that this viability could be due to the selection of stable suppressor mutations re-initiating septation, four microcolonies were isolated from the zone of inhibition of a Δ*sepM* CAS drug strip plate. These colonies were passaged once on drug-free media and fresh conidia were subsequently harvested. Drug strip diffusion assays and CFW staining to detect septa were then repeated. CAS drug strip diffusion assays revealed a similar pattern of patchy growth within a defined zone of inhibition for all four isolates tested (Fig. 7A). This resistance pattern is identical to the Δ*sepM* parental strain and in contrast to the fulminant growth exhibited by the control strain in the presence of drug (Fig. 4), indicating the isolates had not developed wild-type levels of echinocandin resistance. In addition, the ability to form septa was not recovered in any of the four isolates examined (Fig. 7B). Echinocandin stress has been previously shown to transiently induce hyperseptation in *A. fumigatus* (26). In agreement with these reports, we noted that control strain hyphae sampled from the CAS zone of clearance displayed a hyperseptate phenotype (Fig. 7C). Strikingly, the Δ*sepM* microcolonies formed within the CAS zone of clearance maintained the ability to hyperseptate in response to drug stress (Fig. 7D). Therefore, SepM-dependent SIN regulation does not appear essential for the hyperseptation response to echinocandins. We hypothesized this could be due to SepM-independent activity of its putative SIN kinase binding partner, SepL.

### Overexpression of *sepL* in Δ*sepM* modestly recovers the septation defect

**Fig 8:**
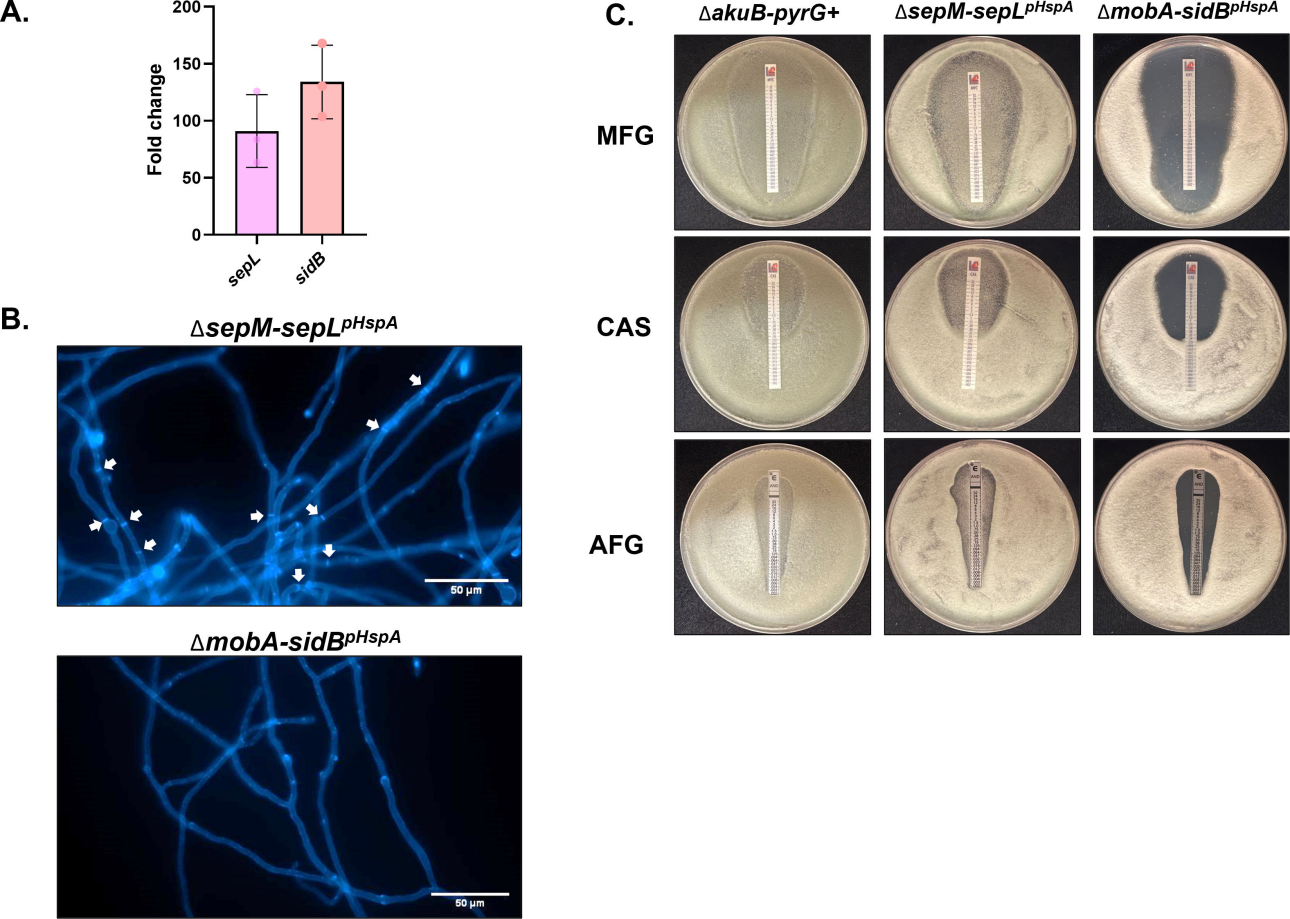
MobA is essential for SidB kinase septation signaling. (**A**) RT-qPCR analysis of expression levels of *sepL* and *sidB* in the *sepL* overexpression mutant (Δ*sepM-sepL^pHspA^*) and the *sidB* overexpression mutant (Δ*mobA-sidB^pHspA^*), respectively, compared to their parent strains. Average data from three biological replicates with two technical replicates for each are shown for each strain. Error bars represent standard deviation. (**B**) Representative micrographs of mature hyphae of the indicated strains stained with CFW to visualize septa. Submerged cultures containing 10^4^ conidia/mL were grown for 16 h at 37°C. Coverslips were removed from culture and stained with CFW before visualization. Septa are indicated by white arrows. Scale bar = 50 µm. (**C**) Drug strip diffusion assays to analyze echinocandin susceptibility. Five-hundred microliters containing 10^6^ conidia of the indicated strains was spread across glucose minimal media agar plates and allowed to dry. Drug strips containing CAS, MFG, or AFG were applied to the plates, which were incubated for 2 days at 37°C. Representative images of three plates per condition are shown.

To test the hypothesis that SepM and MobA are differentially required for the septation-associated functions of their cognate SIN kinases, we generated strains overexpressing *sepL* in the Δ*sepM* mutant (Δ*sepM-sepL^pHspA^*), and overexpressing *sidB* in the Δ*mobA* mutant (Δ*mobA-sidB^pHspA^*) placing both kinase-encoding genes under control of the constitutively active *hspA* promoter (Fig. S1). Expression levels of both kinases in each background was confirmed by reverse transcription-quantitate polymerase chain reaction (RT-qPCR) (Fig. 8A) (30). CFW staining was first employed to examine if overexpression of *sepL* or *sidB* could overcome the loss of septation phenotype in the Δ*sepM* or Δ*mobA* mutants. Whereas the *ΔmobA-sidB^pHspA^* strain did not complement the underlying septation defect, overexpression of *sepL* in the Δ*sepM* background was sufficient to restore hyphal septation (Fig. 8B). Similarly, overexpression of *sidB* was unable to restore echinocandin resistance to the Δ*mobA* mutant (Fig. 8C). Overexpression of *sepL*, however, improved growth of the Δ*sepM* mutant within the zone of clearance for all echinocandins tested (Fig. 8C) when compared to the sepM parental background (Fig. 4A). From these data, we conclude that SepL likely does not depend exclusively on SepM for activation and/or signaling within the SIN pathway, whereas MobA is absolutely required for SidB activation.

### SepM and MobA are required for virulence in a mouse model of IPA

**Fig 9:**
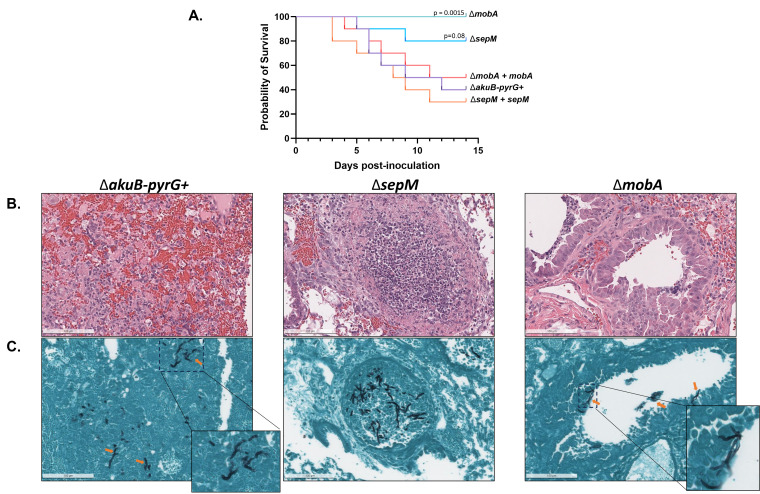
The ∆*mobA* mutant displays significantly reduced virulence in a murine model of invasive aspergillosis. (**A**) Kaplan-Meier survival curves comparing probability of survival of mice inoculated with the indicated strain over 14 days. *N* = 10 mice/group. Data were analyzed by log-rank test. (**B**) Representative images from hematoxylin and eosin-stained tissue sections of mouse lungs harvested 4 days post-infection. (**C**) Representative images from Grocott’s methenamine silver-stained tissue sections of mouse lungs harvested 4 days post-infection. Hyphae are stained black and indicated with orange arrows.

To determine the role of SepM and MobA in virulence, a corticosteroid mouse model of IPA was employed, comparing virulence of the control and manipulated strains (31). One day prior to infection, mice (*n* = 10 per experimental group) were immunosuppressed via subcutaneous injection with triamcinolone acetonide. Mice were inoculated intranasally with 5 × 10^6^ conidia suspended in sterile saline, or sterile saline alone (Sham), and monitored for 14 days (Fig. 9A). Sham treatment resulted in non-significant mortality (data not shown). The control strain induced 60% mortality by day 14. Infection with the Δ*sepM + sepM* and Δ*mobA + mobA* complemented strains similarly resulted in 70% and 50% mortality, respectively (Fig. 9A). These mortality rates were not statistically significantly different compared to the control (log-rank test). The Δ*mobA*-infected mice survived for the duration of the experiment, whereas the Δ*sepM* mutant induced 20% mortality (Fig. 9A). To ensure that mortality was associated with invasive disease progression, mice were treated as above and sacrificed 4 days after infection for histopathological analysis. Lungs were harvested and tissue sections were stained with hematoxylin and eosin (H&E) and Grocott’s methenamine silver (GMS) (Fig. 9B and C). Generalized tissue inflammation was visible in H&E-stained sections from all groups. Invasive hyphae were visible throughout GMS-stained lung sections from mice infected with the control strain. Sporadic tissue invasion was visible in the Δ*sepM* mutant whereas the Δ*mobA-*inoculated sections revealed superficial colonization of the airways with rare tissue invasive growth (Fig. 9C). These data are in agreement with previous findings that *A. fumigatus* septation-deficient mutants suffer from virulence defects (7).

## DISCUSSION

The data reported here represents the first characterization of SIN regulatory components in a human pathogenic filamentous organism. Although some phenotypes reported in this paper are similar to those uncovered from studies in other fungi, such as the conidiation defect which has been identified in septation mutants of *Aspergillus nidulans*, we have noted important differences for *A. fumigatus* (32). For example, the GTPase Spg1 is essential for septation and viability in *S. pombe*, yet Δ*spgA* showed no discernible septation phenotype in our experiments (9). Mutants of Byr4 and Cdc16, the two-component GAP of Spg1, arrest as hyperseptate yeast cells during *S. pombe* cytokinesis. In addition, repression of their orthologs in *A. nidulans* results in modest increases in rate of septation (33). This phenotype was not obvious from our experiments using deletion mutants for the orthologous genes, *byrA* and *bubA*, in *A. fumigatus*. While a modest growth defect was noted in the Δ*byrA* and Δ*bubA* mutants, their impact on morphology was minimal in *A. fumigatus*. The lack of septation deficiency phenotype in Δ*spgA* is especially noteworthy, as it suggests a major departure from the current paradigm of SIN regulation (32, 34–36). Additional studies will be necessary to elucidate the regulatory mechanisms of *A. fumigatus* SepH as the SIN kinase at the head of pathway. In *Sordaria macrospora,* the striatin-interacting phosphatase and kinase complex (STRIPAK) was shown to be involved in negative phospho-regulation of the SepH ortholog, CDC7 (37). The *S. pombe* SIN-inhibitory phosphatase complex, an orthologous complex of STRIPAK, is also inhibitory toward the SIN (38). It is possible that similar mechanisms may be in play for regulation of SIN signaling through SepH in *A. fumigatus*. The STRIPAK has been characterized in *A. nidulans* and is involved in photosensitive metabolism and mitogen activated protein (MAP) kinase signaling (39). *A. nidulans* STRIPAK mutants exhibited conidiation defects and were hypersusceptible to cell wall stress, which are both phenotypes consistent with a septation defect, though a role in septation remains to be investigated.

Our work uncovered a modest but statistically significant increased rate of germination in the ∆*sepM* and ∆*mobA* mutants. In fact, we find that the pre-mature germination phenotype is not merely limited to mutation of these two SIN regulatory components, but is also evident in mutants carrying loss-of-function mutations in the SIN kinases themselves (Fig. S4). The impetus for early germination in the absence of SIN signaling remains unknown. The Morphogenesis Orb6 network (i.e., MOR pathway) has been shown to regulate polarized growth in *S. pombe* and *Neurospora crassa*, and negative crosstalk between the MOR and SIN has been established such that septation and polarized growth do not occur at the same time (34, 40). The MOR pathway is not yet characterized in *A. fumigatus,* but the terminal MOR kinase ortholog, CotA, was identified in the screening of a protein kinase disruption library generated in our laboratory as being important for normal colony development (7). Although septation and mitosis are not essentially coupled in *A. fumigatus* as they are in yeast organisms, decreased SIN activity in our characterized mutants may result in reduced inhibition of the *A. fumigatus* MOR pathway, generating early polarity establishment. If this is the case, it is unclear how an increase in establishment of early growth would lead to the radial growth defect often displayed by septation-deficient strains (7, 41). One explanation may be an increased instance of hyphal tip lysis under normal growth conditions. As we more readily detected PI-positive hyphae under non-stress growth conditions in Δ*sepM* and Δ*mobA*, we hypothesize that the incidence of tip lysis may be increased in aseptate strains. As activation of cell wall biosynthesis machinery for construction of the septal wall is a likely downstream output of the SIN pathway, it is possible that septation-deficient *Aspergilli* display weakened cell wall integrity and therefore may lyse more readily than wild-type hyphae. Connections between the SIN pathway and cell wall biosynthesis and integrity pathways are currently under study.

Septation is paramount to maintain viability under echinocandin stress. Though the differences in septation index and survival under echinocandin stress between Δ*sepM* and Δ*mobA* were not statistically significant, Δ*sepM* was more capable of coping with echinocandin stress throughout the study as evidenced by maintained viability in the face of echinocandin stress on solid agar, production of paradoxical growth at high echinocandin concentrations, and retention of a hyperseptation stress response. These data, combined with our overexpression analyses, suggest that SepL activation or function within the SIN pathway is not entirely dependent on SepM. In contrast, the terminal SIN kinase, SidB, is entirely dependent on the presence of MobA. Therefore, the SidB/MobA complex may be the more relevant target in a search for effective septation inhibitors. Due to the observation that Δ*sepM* was not completely growth inhibited but formed compact microcolonies under echinocandin stress, we hypothesized that genetic adaptations to compensate for the loss of *sepM* may be possible. Though not the case in the present study, suppressor mutations that bypass septation defects have been identified in *A. nidulans* and *N. crassa* (42–44). It is plausible that such mutations are also possible in *A. fumigatus* but did not occur spontaneously as they do in *N. crassa* (44). Identifying potential suppressor mutations could be an important tool for understanding the full repertoire of septation effectors. Novel mechanisms supporting survival under echinocandin stress and novel effectors of tissue invasive growth may also be identified in this way. Our data suggest that SepM is non-essential for either the activation of SepL or for SepL function in the SIN pathway. We also find that the stress adaptation responsible for hyperseptation under caspofungin stress and the paradoxical effect does not require *sepM*, whereas the role for *mobA* is clearly essential (26). More research is required to fully understand this phenomenon in *A. fumigatus* as a potentially important response to cell wall stress.

Taken together, our data further support the SIN pathway as a potential target to be used in combination with echinocandins to improve therapies against invasive aspergillosis. The septation inhibitors diepoxyoctane and hydroxyurea have been shown to increase echinocandin efficacy *in vitro* when conidia are germinated in the presence of those inhibitors (19). Our work with mutants that are genetically defective in septation machinery corroborate these reports. However, successful therapeutic approaches for most invasive *Aspergillus* infections would require application of therapy after infection has initiated and septate hyphae have already formed (4). It remains to be seen if septation inhibitors would have similar synergistic activity with echinocandins in the setting of such pre-existing invasive disease. Regulation of the SIN of filamentous fungi is critically understudied. The downstream targets of the MobA/SidB complex are still largely unknown or uncharacterized in *A. fumigatus*. As our findings indicate a non-essential role for SpgA in septation initiation, the mechanisms of *A. fumigatus* SepH activation also remain unknown. Future studies will be dedicated to delineating the activators and effectors of SIN signaling in *A. fumigatus* and assessing the potential for improving echinocandin activity through blocking septation in the midst of ongoing invasive disease.

## MATERIALS AND METHODS

### Growth conditions

Strains used or generated in this work can be found in Table S1. Conidia used in this study were harvested from glucose minimal media (GMM) agar prepared as described (45). For animal studies, in which high concentrations of conidia were required of poorly conidiating strains, GMM supplemented with 1.2 M sorbitol was used to stimulate conidiation (46). Conidia were harvested from 2- to 4-day-old cultures grown at 37°C. When broth culture was necessary for nucleic acid extraction, conidia were inoculated into GMM and grown for 16 h in a shaking incubator at 37°C/250 RPM. For all assays which required microscopic visualization of conidia, hyphae, or germlings, conidia were grown on glass coverslips pre-sterilized with 100% ethanol, then submerged in liquid minimal media containing 10^4^ conidia/mL. Individual coverslips were inoculated in a 33 mm Petri dish with 4 mL liquid media. Cultures were grown at 37°C for 16 h when mature hyphae were required, or for 6–10 h when germlings were to be visualized (see below).

### Genetic modifications

All oligonucleotides and CRISPR/Cas9 components used for this study purchased from Integrated DNA Techonlogies (IDT) (Coralville, IA, USA) are listed in Table S2. To generate the Δ*sepM* and Δ*mobA* mutants, CRISPR-Cas9-mediated gene deletion was performed as previously described (20). For each transformation, two protospacer adjacent motif (PAM) sites were chosen, one immediately upstream and one downstream of the putative coding sequence for each gene, and custom CRISPR RNAs (crRNAs) were designed to target the adjacent protospacer sequences. Full guide RNAs (gRNA) were assembled *in vitro* using the custom crRNAs and commercially available transactivating CRISPR RNA (tracrRNA) (IDT, Inc.). Repair templates, comprised of a gene cassette encoding hygromycin resistance, were generated by PCR using custom oligonucleotides designed to incorporate 40-basepair microhomology regions on either side of each PAM site to facilitate repair template integration and subsequent gene deletion. Ribonucleoprotein complexes (RNPs) were constructed using commercially available Cas9 (IDT. Inc.) and the *in vitro* assembled gRNA. A modified transformation protocol was used to perform transformations at a final volume or 200 µL, as previously described (7). For transformation, 1–5 × 10^5^ Δ*akuB-pyrG*+ protoplasts were incubated with 5 µL RNP, 900 ng repair template (i.e., HygR cassette), 3 µL 60% polyethylene glycol (PEG) 3350, and 11 µL sorbitol-Tris-calcium chlordie (STC) buffer. This mixture was incubated on ice for 50 min, before addition of 57 µL PEG 3350. Following an additional 20 min of incubation at room temperature, 113 µL STC buffer was added. The entire volume was then plated onto a single agar plate containing GMM + 1.2 M sorbitol. Protoplasts were allowed to recover overnight at room temperature. Top agar media containing 1.2 M sorbitol supplemented with hygromycin (150 µg/mL final concentration) was added and the plate was incubated at 37°C until colonies became visible. Single colonies were picked to GMM agar plates and grown for 3 to 4 days. Conidia were harvested, DNA were extracted, and transformants were screened by diagnostic PCR to confirm successful gene targeting. Further confirmation was obtained via Sanger sequencing. The Δ*spgA*, Δ*byrA,* and Δ*bubA* mutants were generated by an identical process, using protoplasts from the wild-type CEA10 strain instead.

To generate the overexpression of *sepL* and *sidB* in Δ*sepM* and Δ*mobA* backgrounds, transformations were performed as above. For crRNAs used to generate these mutants, one 5´ crRNA was designed to target the nearest protospacer sequence upstream of the initial methionine of *sepL* or *sidB*. Repair templates, comprised of a gene cassette encoding phleomycin resistance followed by the *A. fumigatus hspA* promoter (47), were generated by PCR using the plasmid *pPhleHspA*, built by our research group, as a template. *pPhleHspA* was built by cloning the phleomycin resistance cassette into plasmid *pJMR2* using restriction endonucleases *NotI* and *SpeI*, removing the hygromycin resistance cassette in the process (47). Custom oligonucleotides for amplifying the repair template were designed to integrate 40-basepair microhomology regions to direct homologous integration at the region of the Cas9-induced double-strand break. Resulting mutants were screened as described above, using diagnostic PCR and Sanger sequencing. The overexpression of *sepL* in the Δ*sepM* background and *sidB* in the Δ*mobA* background was confirmed by RT-qPCR compared to their respective parent strains, Δ*sepM* and Δ*mobA*.

For gene complementation, repair templates containing a phleomycin resistance-encoding gene cassette cloned downstream of the gene of interest were generated using plasmid pAGRP (48). The gene of interest was amplified from genomic DNA extracted from CEA10 using gene-specific oligonucleotides designed to integrate *BamH*I and *Not*I restrictions sites and subsequently cloned into pAGRP upstream of the phleomycin resistance cassette using the *BamH*I and *Not*I restriction sites (48). The full repair template was then PCR amplified using oligonucleotides designed to incorporate 40-basepair regions of homology upstream and downstream of a single PAM site selected to ensure integration such that the re-inserted gene is controlled by the endogenous promoter (Fig. S1). Transformations using the deletion mutants as the parent strains were carried out as described above. Gene integration was confirmed by diagnostic PCR and Sanger sequencing.

### RNA extraction and RT-qPCR

RNA extraction and subsequent RT-qPCR were performed as previously described (49). Briefly, 5 × 10^7^ conidia of each overexpression strain (∆*sepM-sepL^pHspA^ and* ∆*mobA-sidB^pHspA^*), and Δ*sepM* and Δ*mobA* as controls, were grown in minimal broth for 16 h at 37°C with agitation at 250 rpm. After the incubation time, the mycelia were harvested and washed with distilled water before lyophilizing overnight. Then, the mycelia were powdered using a mortar and pestle under liquid nitrogen. The powdered mycelia were resuspended in 1 mL of Trizol (Invitrogen, Waltham, MA, USA) and incubated for 5 min on ice. After this incubation time, 200 µL of chloroform-isoamyl alcohol (24:1) (Thermo Scientific, Waltham, MA, USA) was added to each sample. The samples were incubated 3 min on ice and then centrifuged at 13,000 rpm for 15 min. Then, 350 µL of the clear phase was mixed with the same amount of ice-cold 70% ethanol, and all the volume was transferred to the RNAeasy kit (Qiagen, Germantown, MD, USA) columns following manufacturer’s instructions. The RNA samples (6 µg of each) were treated with Turbo DNAse (Invitrogen, Waltham, MA, USA). Finally, 500 ng of RNA were retrotranscribed using the ProtoScript II cDNA synthesis kit (New England Biolabs, Ipswich, MA, USA) following manufacturer’s instructions. Remaining RNA traces were eliminated by treating the resulting cDNA samples with RNAase H (New England Biolabs, Ipswich, MA, USA) following manufacturer’s instructions. The RT-qPCR reactions (20 µL/reaction) were done in a CFX Connect Real-Time System (Bio-Rad, Hercules, CA, USA) using SYBR Green Master Mix (Bio-Rad, Hercules, CA, USA). The reaction conditions were 95°C for 3 min, followed by 36 cycles of 10 s at 95°C, 15 s at 62.5°C, and 45 s at 72°C. In order to check the specificity of the primers, melting curve analysis was run immediately after PCR completion. Primers specific for *sepL*, *sidB,* and the housekeeping gene β-tubulin (*tubA*) were designed in the last exon near the 3´ terminus and used to run the reactions. Cycle threshold values (c_t_) for the targets *sepL* and *sidB* were normalized to *tubA*. Finally, the relative expression was determined using the 2^-ΔCt^ and 2^-ΔΔCt^ method (30). All the assays were carried out in technical and biological triplicates.

### Assay for growth, morphology, and conidiation

To assess colony morphologies, 1,000 conidia were inoculated onto the center of 100 mm Petri dishes with GMM agar and incubated for 96 h at 37°C. Cultures were grown in triplicate for each strain and colony diameter was measured every 24 h. After 96 h, the cultures were harvested in 20 mL sterile water using a sterile cotton swab and filtered through sterile miracloth, and concentration was calculated using a hemocytometer. Total numbers of conidia recovered were normalized to colony area (mm^2^). A two-way analysis of variance (ANOVA) was used (GraphPad Prism) to determine statistical significance of differences in radial growth between strains. One-way ANOVA with Turkey’s test was used to determine statistical significance of difference in conidia per square millimeter after 96 h.

### Germination rate assay

For determination of germination rate, 1 mL of GMM broth containing 5 × 10^4^ conidia of the indicated strain was inoculated into each well of a 24-well plate and plates were incubated at 37°C for. Media was removed from the wells at specific timepoints (6, 8, 10, or 12 h post-inoculation) and replaced with 10% formalin solution to fix the germlings. Germlings were counted directly from the plate using a light microscope at 40× magnification. Conidia were designated as ungerminated, germinated (showing any sign of germ tube formation or polarity establishment, including a tear drop shape), or as multi-germinated, showing established polarity in two or more directions. *P*-values were calculated using a two-way ANOVA with Turkey’s test in GraphPad Prism 9. Germination assays were conducted with three biological replicates per strain, using conidia harvested from three cultures.

### Cell wall and echinocandin stress assays

Susceptibility to cell wall stress was qualitatively assessed by monitoring visible growth in the presence of cell wall stress agents. To do so, 5 µL of a 10^6^ conidia/mL suspension of each strain was spot-inoculated onto media supplemented with increasing concentrations of CR or CFW. Cultures were maintained for 48 h, and susceptibility was determined by the presence or absence of visible growth. Susceptibility to echinocandins was assessed using concentration gradient strips of anidulafungin, caspofungin, and micafungin (Liofilchem or E-test) on minimal media. Five hundred microliters of a 10^6^ conidia/mL suspension was spread evenly over minimal media followed by application of the drug strip after drying. Cultures were grown at 37°C for 48 h and the zone of clearance was assessed. To determine the nature of the echinocandin activity on the mutants (fungicidal or fungistatic), three random cores from the visible zones of clearance on each plate were transferred to drug-free minimal media plates and cultured for an additional 2 days at 37°C. Fungicidal vs fungistatic activity was determined by the absence or presence of visible growth from those cores.

### Fluorescent staining

The fluorescent stains used in this study included CFW, PI, and CFDA and all staining was performed as previously described (7). CFW and PI were used at 12.5 µg/mL final concentration and CFDA was employed at 50 µg/mL. All staining solutions were prepared in PBS. For CFW and PI staining, coverslips with adherent fungus were washed twice with phosphate buffered saline (PBS) pH 7.4 (Gibco) for 5 min, then submerged in staining solution for 5 min. Following this incubation, coverslips were washed twice additionally in PBS for 10 min before being mounted onto glass microscope slides. CFW, PI, and CFDA fluorescence was visualized using 4’,6-diamidino-2-phenylindole (DAPI), tetramethylrhodamine (TRITC), and GFP filters, respectively. Fluorescence microscopy was conducted with a Nikon TI2-A inverted microscope equipped with a Prime BSI express monochrome camera or a Nikon NiU microscope equipped with a Nikon DS-Qi1Mc camera.

### Mouse model of invasive aspergillosis

Survival studies were conducted as previously described (31). Briefly, 6-week-old CD-1 female mice (Charles River) were immunosuppressed with 40 mg/kg triamcinolone acetonide (Kenalog, Bristol-Myers Squibb, Princeton, NJ, USA), administered via subcutaneous injection on day –1 relative to infection. Mice were infected intranasally with 5 × 10^6^ conidia suspended in 30 µL sterile injectable water. The sham group was inoculated with sterile injectable water alone. Survival was monitored for 14 days following infection. Statistics were performed using the log-rank test on GraphPad Prism 9. Group size was equal to 10 mice per group. To histologically assess tissue invasion, mice were immunosuppressed and infected as described above. Mice were sacrificed on day +4 and lungs were harvested and fixed in 10% buffered formalin. Fixed lung samples were paraffin-embedded, sectioned, and mounted for staining using Grocott’s methenamine silver stain (HistoWiz).

## SUPPLEMENTAL MATERIAL

Figure S1 msphere.00695-23-s0001.tif Schematic for gene manipulations.

Figure S2 msphere.00695-23-s0002.tif Analysis of conidial pigmentation.

Figure S3 msphere.00695-23-s0003.tif Analysis of upstream SIN genes.

Figure S4 msphere.00695-23-s0004.tif Germination data for SIN pathway mutants.

Supplemental Legends msphere.00695-23-s0005.docx Legends for supplemental figures and tables.

Tables S1 and S2 msphere.00695-23-s0006.xlsx Strains and primers used in this study.

ASM does not own the copyrights to Supplemental Material that may be linked to, or accessed through, an article. The authors have granted ASM a non-exclusive, world-wide license to publish the Supplemental Material files. Please contact the corresponding author directly for reuse.
