# Supplementary figures and images for: MOB-mediated regulation of septation initiation network (SIN) signaling is required for echinocandin-induced hyperseptation in Aspergillus fumigatus
Source: mSphere. 2024 Feb 13;9(3):e00695-23. doi: 10.1128/msphere.00695-23 (PMC10964416; doi:10.1128/msphere.00695-23)

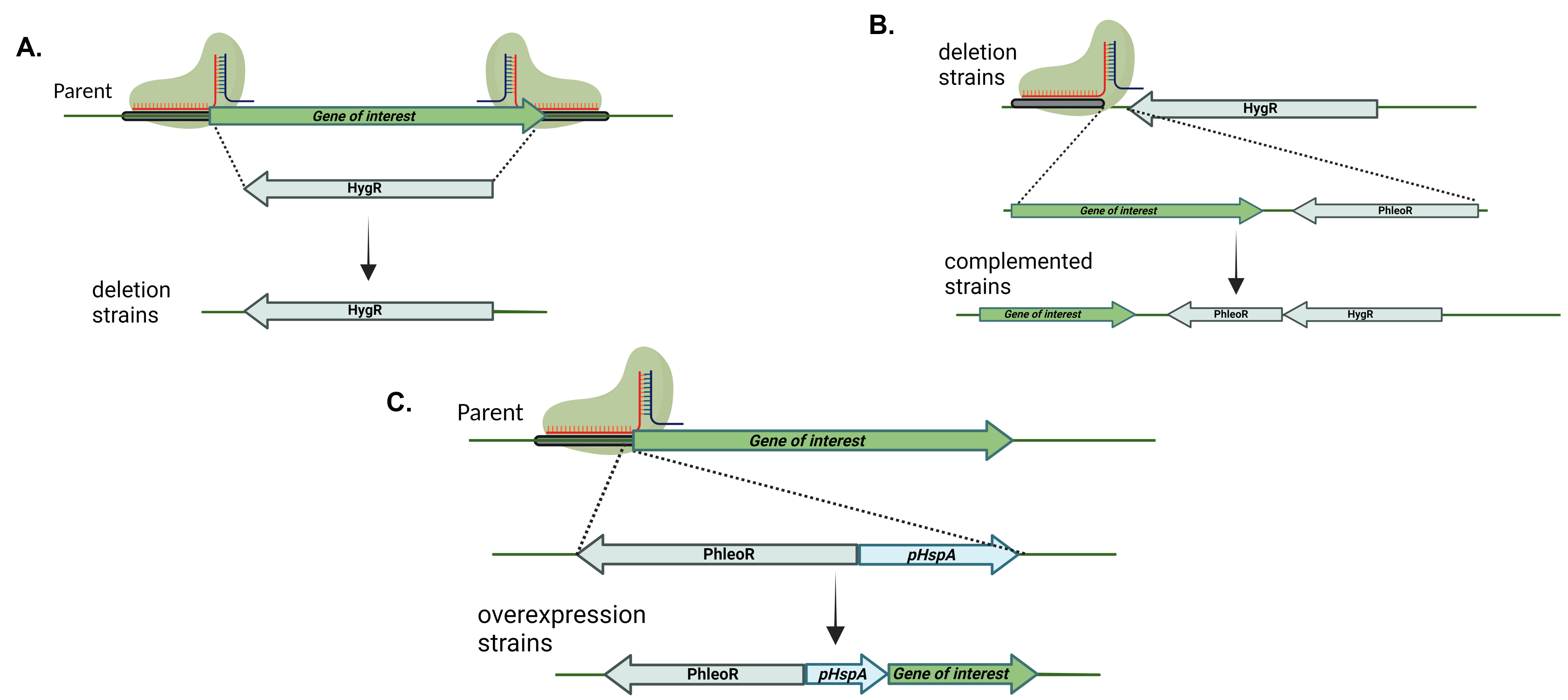

Supplement: Figure S1 — Schematic for gene manipulations. [file msphere.00695-23-s0001.tif]

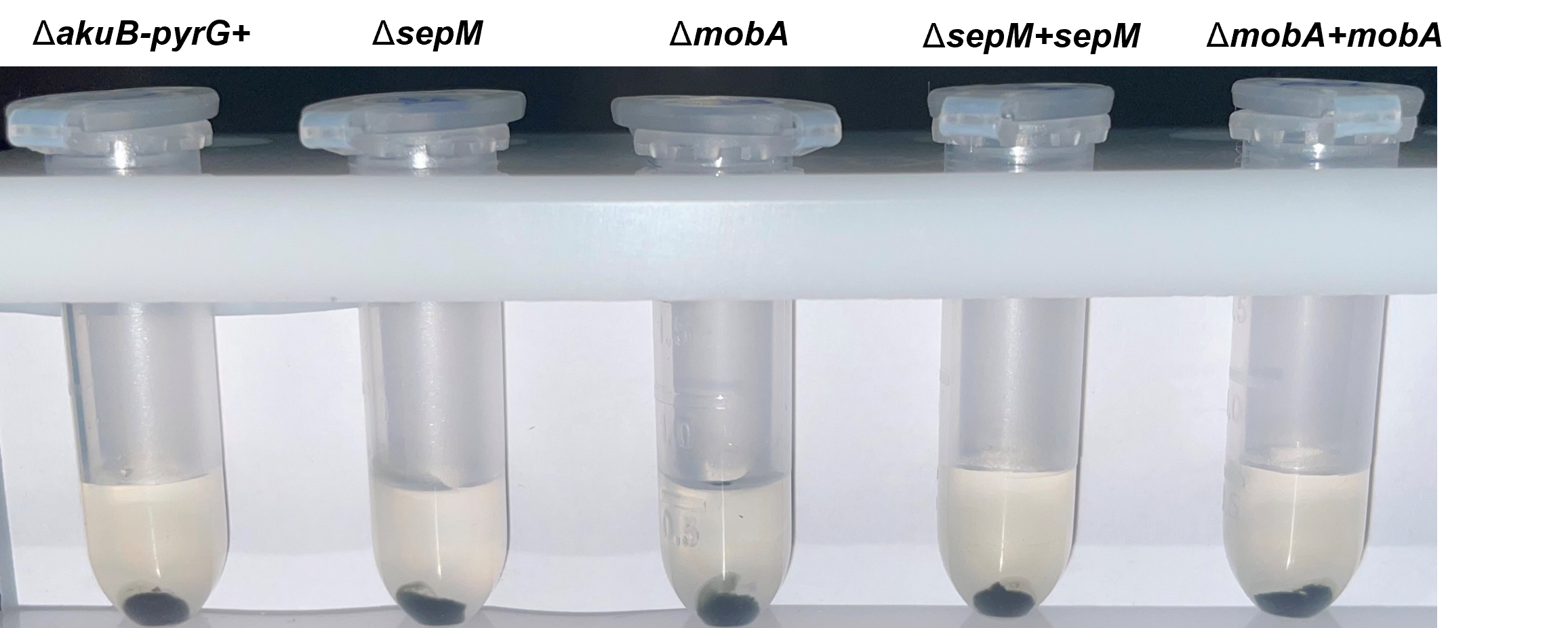

Supplement: Figure S2 — Analysis of conidial pigmentation. [file msphere.00695-23-s0002.tif]

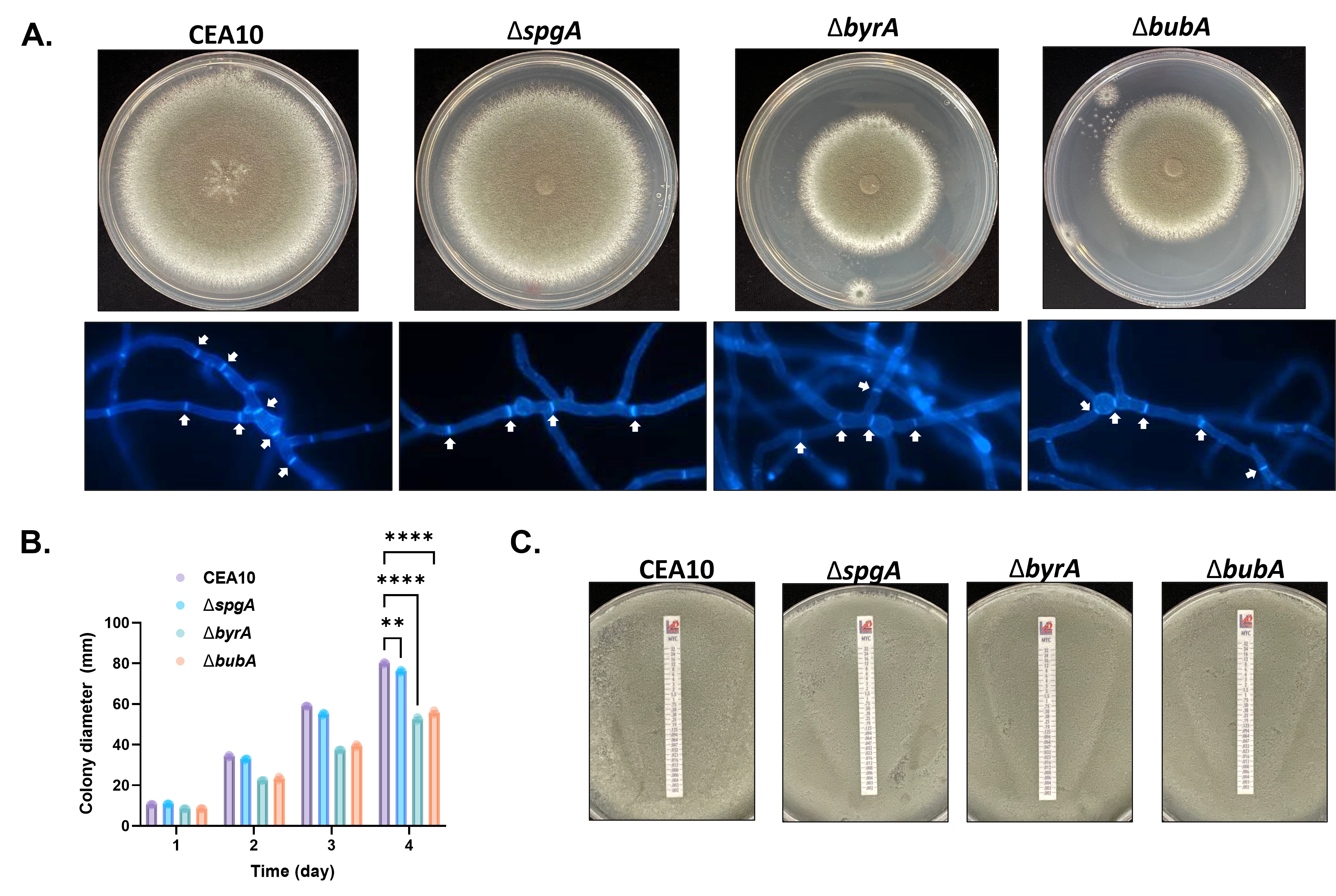

Supplement: Figure S3 — Analysis of upstream SIN genes. [file msphere.00695-23-s0003.tif]

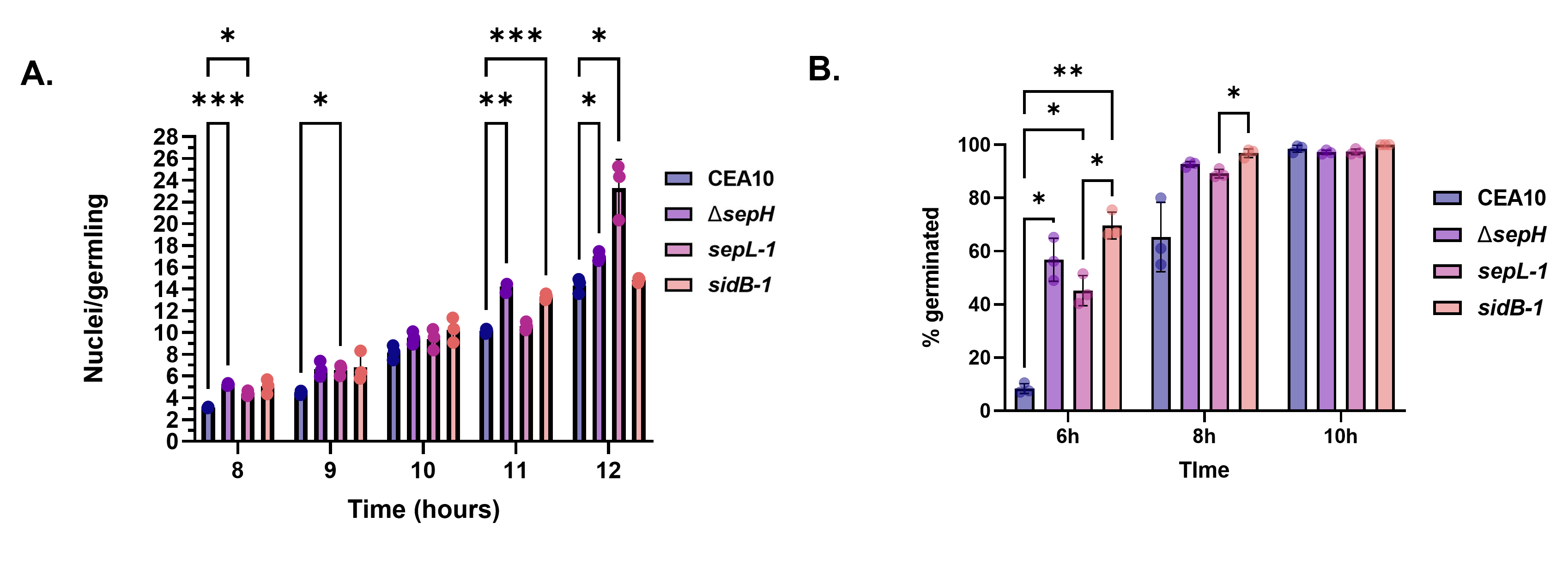

Supplement: Figure S4 — Germination data for SIN pathway mutants. [file msphere.00695-23-s0004.tif]
