# Supplementary material for: MOB-mediated regulation of septation initiation network (SIN) signaling is required for echinocandin-induced hyperseptation in Aspergillus fumigatus
Source: mSphere. 2024 Feb 13;9(3):e00695-23. doi: 10.1128/msphere.00695-23 (PMC10964416; doi:10.1128/msphere.00695-23)
Supplement: Supplemental Legends — Legends for supplemental figures and tables. [file msphere.00695-23-s0005.docx]

**Supplemental Figure 1. Schematics of genetic manipulations. A)** Schematic depicting generation of gene deletion mutants. Gene deletions were performed by replacing the entire gene coding sequence with a hygromycin resistance cassette (HygR) using CRISPR-Cas9 gene editing approaches. **B)** Schematic depicting generation of gene-complemented strains. Complemented strains were generated by re-incorporating the gene coding sequence, coupled to a phleomycin resistance cassette (PhleoR), downstream of the endogenous promoter at each locus. **C)** Schematic depicting generation of overexpression strains. Overexpression strains were generated by inserting a construct containing the PhleoR cassette, flanked by the strong constitutive *hspA* promoter (*pHspA*), immediately upstream of the gene of interest. Schematics were made using Biorender.

**Supplemental Figure 2. Assessment of pigmentation of ∆*sepM* and ∆*mobA.*** Strains were grown for 3 days on GMM agar supplemented with 1.2M sorbitol to induce conidiation from mutant strains. In a 2ml microcentrifuge tube, 1.5 * 10^8^ conidia of the indicated strains were resuspended in 600μl of sterile water and centrifuged at 13,300 RPM for 3 minutes. A photograph of the tubes containing pelleted conidia is shown.

**Supplemental Figure 3. Phenotypic characterization of ∆*spgA*, ∆*byrA* and ∆*bubA*. A)** Colony morphology and septum visualization of the control strain (CEA10), the *spgA* deletion (∆*spgA*), the Δ*byrA* deletion (∆byrA), and the *bubA* deletion (∆bubA). Ten-thousand conidia were spot-inoculated onto the center of GMM agar and allowed to grow for 4 days at 37°C. To visualize septa, conidia from each strain were inoculated over sterile coverslips submerged in minimal media broth and incubated for 10 h at 37°C. After incubation, coverslips were removed and adhered germlings stained with calcofluor white for visualization of septa. White arrows denote septa. Scale bars = 50 µm. **B)** Quantitation of colony diameter for each day post-inoculation. Colony diameter was measured at the end of each 24-hour period. Data presented are the average of three biological replicates per strain ± standard deviation. Data were analyzed by two-way ANOVA with Turkey’s multiple comparisons test. ** = p < 0.01. **** = p < 0.0001. **C)** Drug strip diffusion assays to analyze echinocandin susceptibility. Five-hundred microliters of sterile water containing 10^6^ conidia of the indicated strains were spread across GMM agar plates and allowed to dry. Drug strips containing MFG were applied to the plates, which were incubated for 2 days at 37°C. Representative images of 3 plates per strain are shown.

**Supplemental Figure 4.** **Analysis of mitotic and germination rate during early development for SIN kinase mutants. A)** Quantitation of nuclei per germling during early growth in the wild type (CEA10), Δ*sepH*, *sepL-1*, and *sidB-1* mutants. Each strain was assayed in triplicate for the indicated timepoints. After the indicated time post-inoculation, coverslip cultures were washed, fixed, and stained with propidium iodide to visualize nuclei. Between 60-100 germlings were evaluated for number of nuclei. The average number of nuclei per germling are shown. Data were analyzed by two-way ANOVA using Dunnett’s multiple comparisons test. * = p < 0.05. ** = p < 0.01. *** = p < 0.001. **B**) Analysis of germination rate among the wild type (CEA10), Δ*sepH*, *sepL-1*, and *sidB-1* mutants. Coverslip cultures were grown for each strain in triplicate for the indicated time. At each time point, coverslips were mounted and visualized by light microscopy. Between 200-400 conidia and/or germlings were evaluated for polarity establishment. The average number of cells with established polarity, expressed as percent, is shown. Data were analyzed by two-way ANOVA with Turkey’s multiple comparisons test. * = p < 0.05. ** = p < 0.01.

**Supplementary Table 1. Strains Used in this Study.**

**Supplementary Table 2. Primers and CRISPR/Cas9 components used in this study.**
